# Perifollicular fibrosis and inflammation in androgenetic alopecia and seborrheic dermatitis: diagnostic challenges in differentiation from fibrosing alopecia in a pattern distribution^؆^

**DOI:** 10.1016/j.abd.2026.501408

**Published:** 2026-07-03

**Authors:** Tatiane Elen De Souza, Almut Böer-Auer, Betina Werner

**Affiliations:** a Postgraduate Program, Internal Medicine and Health Sciences, Universidade Federal do Paraná, Curitiba, PR, Brazil; b Dermatologikum Hamburg, Hamburg, Germany; c University of Münster, Münster, NRW, Germany; d Department of Pathology, Universidade Federal do Paraná, Curitiba, PR, Brazil

**Keywords:** Androgenetic alopecia, Dermatitis, seborrheic, Fibrosis, Inflammation, Cicatricial alopecia

## Abstract

**Background:**

Androgenetic Alopecia (AGA) and Seborrheic Dermatitis (SD) are scalp conditions that may show histopathological features overlapping with Fibrosing Alopecia in a Pattern Distribution (FAPD), potentially leading to diagnostic confusion between non-scarring and scarring alopecias.

**Objective:**

To identify histopathological features that distinguish AGA from SD and to evaluate findings overlapping with those described in FAPD, focusing on perifollicular fibrosis and inflammation.

**Methods:**

Transverse scalp biopsy sections from 56 Caucasian male patients (21 AGA, 35 SD) were blindly evaluated. Quantitative follicular parameters and qualitative epidermal, follicular, inflammatory, and adnexal features were assessed and statistically compared.

**Results:**

AGA showed higher vellus follicle counts (p = 0.029) and lower terminal follicle and anagen hair counts (p = 0.002; p = 0.045), confirming follicular miniaturization. Perifollicular fibrosis (infundibular and/or isthmic) was frequent in both conditions, limiting its specificity for scarring alopecias. Mild basal vacuolization (14.3%; p = 0.048) and eccrine duct dilatation (23.8%; p = 0.006) occurred exclusively in AGA, whereas polytrichia was observed only in SD (17.1%; p = 0.050). Perifollicular inflammation was common and non-specific in both groups, occasionally mimicking lichenoid patterns. Demodex spp. was more prevalent in AGA (p = 0.007). Necrotic keratinocytes were rare, with no significant differences.

**Study limitations:**

The study included only male patients and had a limited sample size, a cross-sectional design, and no FAPD comparison. Nonetheless, substantial histopathological overlap exists between AGA, SD, and features attributed to FAPD, particularly perifollicular fibrosis and inflammation. Careful clinicopathological correlation is essential to avoid misclassification, with prognostic and therapeutic implications.

## Introduction

Androgenetic Alopecia (AGA) is primarily diagnosed clinically. Trichoscopy typically demonstrates hair shaft diameter variability and yellow dots.^1^, ^2^, ^3^, ^4^, ^5^, ^6^, ^7^ Histopathologically, AGA is characterized by follicular miniaturization associated with variable degrees of perifollicular lymphocytic inflammation and fibroplasia.^7^, ^8^, ^9^ Although these inflammatory and fibrotic changes are usually mild, they may overlap with features traditionally associated with scarring alopecias, particularly when interpreted in isolation.

Fibrosing Alopecia in a Pattern Distribution (FAPD) has classically been regarded as a variant of Lichen Planopilaris (LPP), presenting with patterned hair loss involving the central scalp and vertex.^10^, ^11^ Clinically, patients may report pruritus or trichodynia and show perifollicular erythema, hyperkeratosis, loss of follicular openings, tufting, and broken hairs. Histologically, FAPD combines features of AGA, such as follicular miniaturization, with perifollicular lichenoid inflammation and fibrosis typical of LPP.^12^, ^13^ Nevertheless, the nosologic status of FAPD remains controversial. Recent studies have questioned its classification as a true variant of LPP, proposing instead that it represents a clinicopathologic pattern characterized by features of diffuse alopecias accompanied by perifollicular inflammation and fibrosis.^14^

Seborrheic Dermatitis (SD) of the scalp, although not primarily an alopecia, may further complicate this diagnostic spectrum. SD is a chronic, relapsing inflammatory dermatosis that may coexist with or exacerbate other hair disorders, including telogen effluvium and AGA.^15^, ^16^ Trichoscopic findings typically include yellow-white scales and arborizing vessels, while histopathology may demonstrate psoriasiform epidermal hyperplasia, parakeratosis, spongiosis, and inflammatory changes involving follicular ostia.^17^ Despite its frequent coexistence with AGA, with a reported prevalence of approximately 27.9%,^18^ the histopathological impact of SD on the hair follicle, particularly regarding perifollicular inflammation and fibrosis, remains insufficiently explored.

In this context, differentiating non-scarring conditions such as AGA and SD from FAPD based solely on histopathological findings may be particularly challenging. Certain features commonly associated with cicatricial alopecias, such as perifollicular fibrosis, basal layer vacuolization, or lichenoid-appearing inflammation, may also be encountered in non-scarring disorders, increasing the risk of misdiagnosis.

This study uses transverse scalp biopsy sections to systematically compare the histopathological features of AGA and SD, with special emphasis on perifollicular fibrosis and inflammation that may mimic the changes described in FAPD. By clarifying areas of overlap and distinction among these entities, this work aims to support more accurate clinicopathological correlation and reduce the risk of misclassifying common non-scarring scalp disorders as scarring alopecias, which carry markedly different prognostic and therapeutic implications.

## Materials and methods

This descriptive, cross-sectional study evaluated Caucasian male patients aged 18–65 years with clinically confirmed SD or AGA at a university hospital’s dermatology outpatient service. Participants, randomly selected based on unambiguous clinical and trichoscopic diagnoses, had SD diagnosed via scalp erythema and fine, white interfollicular scaling in mild cases or intense erythema with coarse, yellowish scaling in severe cases, per established criteria. AGA was diagnosed using the Hamilton-Norwood classification (stages I–IV), characterized by symmetrical, progressive hair shaft thinning in the parietal and vertex regions with occipital area preservation, showing trichoscopic features like hair shaft diameter variability and yellow dots. Exclusion criteria encompassed prior treatment within six months, including systemic or topical medications, microneedling, mesotherapy, or laser therapies, as well as other alopecias, scalp disorders such as atopic dermatitis, contact dermatitis, tinea capitis, or psoriasis, systemic conditions like HIV or neurological disorders, including cerebrovascular disease or Parkinson’s disease, and hypersensitivity to 2% lidocaine used for biopsies. All participants provided written informed consent, and the study was approved by the institutional review board (Research Ethics Committee, from Hospital de Clínicas [Federal University of Paraná], Curitiba – Paraná. Certificate of Presentation for Ethical Appreciation [CAAE 75757223.4.0000.0096] approval nº6.624.101).

Each participant underwent a single 4-mm punch biopsy from the vertex scalp, fixed in formalin, and processed for transverse histological sectioning with hematoxylin and eosin staining. Slides, anonymized with preassigned codes, were evaluated in a blinded manner across all histological levels from epidermis to hypodermis, assessing 35 parameters, including seven quantitative measures, such as total follicular units, total hair follicles, terminal follicles, vellus follicles, follicles in anagen and telogen phases, and sebaceous gland size, defined as the maximal uninterrupted diameter of the largest lobe, and 28 qualitative measures, such as epidermal and follicular spongiosis, defined as edema within epithelial layers, intrafollicular parakeratosis at the ostium or upper follicular canal, basal layer spongiosis/vacuolization, necrotic keratinocytes, microbial elements like yeasts, bacterial colonies, or Demodex spp. in follicular ostia or sebaceous ducts, inflammatory features including perifollicular inflammation, eosinophils, plasma cells, acute neutrophilic folliculitis, foreign body-type granulomatous reactions, and structural or adnexal changes like infundibular dilatation, cicatricial fibrous tracts, perifollicular fibrosis, sebaceous gland atrophy, polytrichia (5 or more hair shafts in the same follicular canal) and eccrine ductal dilatation. For cases with perifollicular inflammation, additional details included affected follicle type, whether terminal or vellus, anatomical location, either isthmus or infundibulum, and lichenoid features, defined as obscuration of the follicular dermoepidermal junction, significant basal vacuolization and lymphocyte exocytosis, and keratinocyte necrosis (necrotic keratinocytes and perifollicular fibrosis further classified by anatomical level and follicle type).

Histopathological data were recorded in Microsoft Excel for analysis, with quantitative variables summarized using mean, standard deviation, and range, and categorical variables analyzed via frequency distributions in one- and two-way contingency tables. Data normality, assessed using the Shapiro–Wilk test, guided the selection of parametric tests, such as *t*-tests, or non-parametric tests, such as Mann-Whitney *U*-tests, for group comparisons, with significance set at p < 0.05. Statistical analyses, performed using Statistica software, compared microscopic findings between SD and AGA groups based on tabulated results.

## Results

**Fig. 1:**
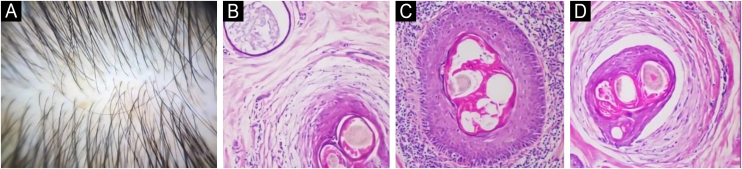
This figure presents a clinical picture and photomicrographs of patients from the androgenetic alopecia group. (A) Trichoscopic view of Patient 58 showing hair shaft diameter variability. (B) Corresponding transverse histological section from Patient 58 showing perifollicular fibrosis, lymphocytic infiltrate, and spongiosis in the follicular epithelium with lymphocyte exocytosis; a dilated eccrine gland is visible in the upper left corner (Hematoxylin & eosin, 20x). (C) Severe lymphocytic inflammation surrounding a hair follicle with mild epithelial spongiosis in Patient 15 (Hematoxylin & eosin, 20x). (D) Prominent perifollicular fibrosis, epithelial thinning, and lymphocytic infiltration in the adjacent dermis in Patient 28 (Hematoxylin & eosin, 20x).

**Fig. 2:**
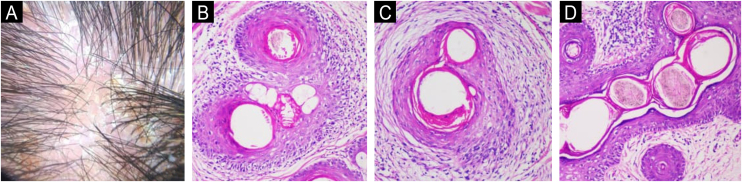
This figure presents a clinical picture and photomicrographs of patients from the seborrheic dermatitis group. (A) Trichoscopic view of Patient 32 showing perifollicular and interfollicular yellowish-white scales. (B) Transverse histological section from Patient 32 depicting the isthmus area of a follicle surrounded by lymphocytic inflammation, with epithelial spongiosis, vacuolar interface change, and lymphocyte exocytosis (Hematoxylin & eosin, 20x). (C) Patient 53 showing prominent perifollicular fibrosis, blurred interface, and lymphocytic infiltration in the follicular epithelium (Hematoxylin & eosin, 20x). (D) Histological section from Patient 30 showing multiple hair shafts in a common follicular canal (polytrichia) (Hematoxylin & eosin, 20x).

Transverse scalp biopsy specimens from 56 Caucasian male participants aged 18–65 years, comprising 35 (62.5%) with Seborrheic Dermatitis (SD) and 21 (37.5%) with AGA, were histologically evaluated, with mean age showing no significant difference between groups (p = 0.16) (Fig. 1, Fig. 2).

Quantitative analysis revealed that SD had significantly higher mean counts of total hair follicles (p = 0.047), terminal follicles (p = 0.002), and anagen hairs (p = 0.045) compared to AGA, which exhibited a significantly greater number of vellus follicles (p = 0.029), consistent with follicular miniaturization in AGA. The anagen-to-telogen ratio was lower in AGA, approaching statistical significance (p = 0.051), while other quantitative measures, such as follicular units, telogen hairs, and sebaceous gland size, defined as the maximal uninterrupted diameter of the largest lobe, showed no significant differences (p > 0.05).

Qualitative analysis identified significant differences favoring AGA for Demodex spp. presence in the follicular canal (42.9% vs. 11.4%; p = 0.007) and sebaceous duct (38.1% vs. 8.6%; p = 0.007), basal layer spongiosis/vacuolization (14.3% vs. 0%; p = 0.048), and eccrine ductal dilatation (23.8% vs. 0%; p = 0.006). Perifollicular fibrosis was prevalent in both conditions, observed in over 60% of cases, with isthmic fibrosis more frequent in AGA (38.1% vs. 14.3%; p = 0.044) and trends toward higher infundibular fibrosis (42.9% vs. 20.0%; p = 0.067) and fibrosis around terminal follicles (p = 0.067) in AGA, while fibrosis around vellus follicles was rare and not significantly different (p = 0.14). Perifollicular inflammation was observed in over 70% of AGA and 60% of SD cases, with no statistically significant difference between the conditions. Necrotic keratinocytes were uncommon, with no significant differences in the infundibulum (p = 0.61) or isthmus (p = 0.27) between groups. Polytrichia, observed exclusively in SD (17.1%), approached significance (p = 0.050). Other qualitative parameters, including epidermal and follicular spongiosis, intrafollicular parakeratosis, bacterial or fungal colonization, perifollicular inflammation, and sebaceous gland size, showed no significant differences (p > 0.14). Histopathologic findings are summarized in Table 1.

**Table 1:** Comparison of 7 quantitative and 28 qualitative histopathologic parameters between patients with androgenetic alopecia (n = 21) and seborrheic dermatitis (n = 35).

Histopathologic Parameters	AGA (n = 21)	SD (n = 35)	p-value
Quantitative parameters			
Total follicular units	14.24 (3.64)	13.82 (4.53)	0.72
Total hair follicles	35.30 (11.38)	42.12 (14.27)	***0.047***
Terminal follicles	24.35 (11.98)	34.29 (12.41)	***0.002***
Vellus follicles	10.95 (6.06)	7.79 (4.97)	***0.029***
Anagen hairs	33.45 (11.68)	40.53 (14.27)	***0.045***
Telogen hairs	1.75 (2.07)	1.29 (2.15)	0.49
Sebaceous gland size (mm)	0.53 (0.07)	0.51 (0.15)	0.27
Qualitative parameters			
Epidermal spongiosis	4 (19.0%)	12 (34.3%)	0.22
Follicular spongiosis	16 (76.19%)	24 (68.57%)	0.54
Intrafollicular parakeratosis	12 (57.1%)	23 (65.7%)	0.52
Basal layer vacuolization	3 (14.3%)	0 (0.0%)	***0.048***
Necrotic keratinocytes – infundibulum	1 (4.8%)	1 (2.9%)	0.61
Necrotic keratinocytes – isthmus	3 (14.3%)	2 (5.7%)	0.27
Yeasts	0 (0.0%)	1 (2.9%)	0.43
Demodex – follicular canal	9 (42.9%)	4 (11.4%)	***0.007***
Demodex – sebaceous duct	8 (38.1%)	3 (8.6%)	***0.007***
Bacterial colonies	15 (71.4%)	21 (60.0%)	0.39
Perifollicular inflammation – infundibulum	16 (76.2%)	22 (62.9%)	0.30
Perifollicular inflammation – isthmus	15 (71.4%)	21 (60.0%)	0.39
Inflammation in terminal hair	17 (81.0%)	24 (68.6%)	0.31
Inflammation in vellus hair	8 (38.1%)	7 (20.0%)	0.14
Lichenoid-type inflammation	0 (0.00%)	0 (0.00%)	0.00
Eosinophils	0 (0.00%)	0 (0.00%)	0.00
Plasma cells	0 (0.00%)	0 (0.00%)	0.00
Acute neutrophilic folliculitis	0 (0.00%)	0 (0.00%)	0.00
Foreign body-type granulomatous reaction	0 (0.00%)	0 (0.00%)	0.00
Infundibular dilatation	6 (28.6%)	15 (42.9%)	0.28
Cicatricial fibrous tracts	1 (4.8%)	0 (0.0%)	0.38
Perifollicular fibrosis – infundibulum	9 (42.9%)	7 (20.0%)	0.067
Perifollicular fibrosis – isthmus	8 (38.1%)	5 (14.3%)	***0.044***
Fibrosis around terminal follicles	9 (42.9%)	7 (20.0%)	0.067
Fibrosis around vellus follicles	2 (9.5%)	0 (0.0%)	0.14
Sebaceous gland atrophy	0 (0.0%)	0 (0.0%)	0.00
Polytrichia	0 (0.0%)	6 (17.1%)	***0.050***
Eccrine duct dilatation	5 (23.8%)	0 (0.0%)	***0.006***

AGA, Androgenetic Alopecia; SD, Seborrheic Dermatitis.

Quantitative parameters are presented as mean (standard deviation) and qualitative parameters as n (%). p-values were obtained using appropriate statistical tests based on variable type and data distribution.

## Discussion

The diagnosis of diffuse alopecia often presents clinical and histopathologic challenges. Fibrosing Alopecia in a Pattern Distribution (FAPD) is traditionally regarded as a variant of Lichen Planopilaris (LPP), characterized by diffuse central scalp involvement and overlapping features of Androgenetic Alopecia (AGA) and classic LPP.^19^, ^20^, ^21^, ^22^, ^23^ However, recent viewpoints suggest that FAPD may not represent a distinct nosologic entity, but rather a clinicopathologic pattern encompassing early or diffuse LPP, fibrosing AGA, and AGA associated with inflammatory scalp conditions such as Seborrheic Dermatitis (SD).^14^, ^24^, ^25^ Its classification as a primary cicatricial alopecia therefore remains debated,^26^ particularly in light of the growing recognition of “scarring AGA” in the literature.^27^ Notably, previous reviews, including that of Griggs et al.,^3^ have not systematically examined the histopathologic criteria that distinguish AGA with perifollicular fibrosis from FAPD.

Although AGA is not classically defined as an inflammatory or scarring alopecia, chronic cases frequently exhibit mild perifollicular lymphocytic inflammation and fibrosis.^28^, ^29^, ^30^ This inflammatory component has been proposed as a contributor to disease progression, although it differs in intensity and distribution from the dense lichenoid infiltrates characteristic of LPP, a progressive cicatricial alopecia that may also present diffusely.^30^ In the present study, perifollicular fibrosis was commonly observed in both AGA and SD, but was more prevalent in AGA. Isthmic fibrosis was significantly more frequent in AGA (38.1%) than in SD (14.3%; p = 0.044), while infundibular fibrosis occurred in 42.9% of AGA cases versus 20.0% of SD cases (p = 0.067). In both groups, fibrosis predominantly involved terminal follicles. These findings reinforce that perifollicular fibrosis, when considered in isolation, lacks specificity for the diagnosis of scarring alopecias.

Other inflammatory features, including perifollicular inflammation involving terminal and vellus follicles, follicular spongiosis, and epidermal spongiosis, were frequently observed in both groups without significant differences. Basal layer vacuolization was identified exclusively in AGA cases (14.3%; p = 0.048), potentially reflecting chronic follicular injury associated with long-standing disease. Necrotic keratinocytes were uncommon in both groups and did not reach statistical significance. Importantly, basal vacuolization and necrotic keratinocytes are classically associated with cicatricial alopecias such as LPP and Frontal Fibrosing Alopecia (FFA).^31^, ^32^, ^33^, ^34^, ^35^, ^36^ Their occasional presence in non-scarring conditions such as AGA and SD reveals a key diagnostic pitfall and highlights the risk of misclassifying these entities as FAPD.

Misdiagnosis in this context carries important clinical consequences. Erroneously labeling common non-scarring conditions as cicatricial alopecias implies a poor and irreversible prognosis, may increase patient anxiety, and can lead to inappropriate therapeutic decisions. This risk is further amplified when AGA and SD coexist, a frequent clinical scenario, as the combination of follicular miniaturization, perifollicular inflammation, and fibrosis may falsely suggest a scarring process. The overlapping histopathologic features observed in this study, when contextualized with those reported for FAPD in the literature, are summarized in Table 2.^10^, ^11^, ^23^, ^32^^,^^37^

**Table 2:** Comparison of histopathological features in androgenetic alopecia, seborrheic dermatitis, and fibrosing alopecia in a pattern distribution (literature-based).

Histopathological Parameter	AGA (n = 21)	SD (n = 35)	FAPD
Follicular Miniaturization	+++ (p = 0.029, significantly higher vellus follicles)	− (0%)	++ (∼70%)
Perifollicular Fibrosis	+ (∼40% overall; 38.1% isthmic, 42.9% infundibular)	+/- (∼17%; 14.3% isthmic, 20.0% infundibular)	+++ (79–93% of cases)
Perifollicular Inflammation	++ (> 70%)	++ (> 60%)	+++ (55–100%, often lichenoid)
Basal Layer Vacuolization	+/- (14.3%)	− (0%)	++ (∼57%, associated with interface changes)
Necrotic Keratinocytes	+/− (Uncommon, ∼10%)	+/− (Uncommon, ∼10%)	+ (29%)
Polytrichia	− (0%)	+/− (17.1%)	+ (Exact frequency varies; reported in LPP variants)
Eccrine Duct Dilatation	+/− (23.8%)	− (0%)	+ (Exact frequency varies; reported in LPP variants)
Demodex Presence	+ (∼40%)	+/− (∼10%)	(Not reported in LPP/FAPD literature)
Yeasts of Malassezia	− (0%)	+/− (∼2.8%)	+/− (∼1.5%)

AGA, Androgenetic Alopecia; FAPD, Fibrosing Alopecia in a Pattern Distribution; LPP, Lichen Planopilaris; SD, Seborrheic Dermatitis.

+++: > 75% of cases; ++: 50%–75% of cases; +: 25%–49% of cases; +/−: 1%–24% of cases; −: 0% of cases.

A distinctive finding in this study was the presence of eccrine duct dilatation, observed exclusively in AGA cases (23.8%; p = 0.006). Previous studies have shown that eccrine duct dilatation is more commonly encountered in lymphocytic cicatricial alopecias,^38^ is less frequent in non-scarring alopecias, and is particularly prominent in neutrophilic scarring subtypes.^39^ In scarring alopecias, this change is thought to result from dermal fibrosis and ductal obstruction, whereas in non-scarring disorders, it may represent a reactive change related to a chronic inflammatory microenvironment.^39^ In only one AGA case, a fibrous scar tract was identified, likely representing follicular “dropout” occasionally seen in advanced stages of the disease rather than true cicatricial alopecia.

An unexpected and novel observation was the presence of polytrichia, identified exclusively in SD cases (17.1%; p = 0.050). According to Griggs et al.,^3^ perifollicular casts surrounding a tuft of emerging hairs are considered a characteristic feature of FAPD and often guide biopsy site selection. Although polytrichia has traditionally been associated with scarring alopecias such as LPP, its occurrence in SD suggests that this feature may also arise in non-scarring inflammatory scalp conditions, potentially contributing to diagnostic ambiguity.

Taken together, these findings support the hypothesis proposed by Werner et al.,^14^ suggesting that FAPD may represent a shared histopathologic pattern observed in early LPP, fibrosing AGA, and inflammatory scalp disorders such as SD, often superimposed on AGA, as summarized by comparison with published FAPD data (Table 2). This interpretation is further supported by the recognized overlap between FAPD and diffuse variants of LPP, including LPP in a pattern distribution and other scarring patterns of hair loss, which share clinical, trichoscopic, and histologic features.^3^, ^11^, ^14^, ^32^

As expected, follicular counts confirmed that SD, although an inflammatory scalp condition, is not intrinsically associated with alopecia. Patients with SD demonstrated higher total follicle counts and a predominance of terminal hairs, whereas AGA was characterized by a significantly greater proportion of vellus follicles and reduced terminal hair counts, consistent with follicular miniaturization. These quantitative findings allowed a clear histopathologic distinction between AGA and SD, validating the appropriateness of clinical stratification and reinforcing that diagnostic overlap arises primarily from inflammatory and fibrotic features rather than from follicular architecture.

Regarding microbial findings, no statistically significant differences were observed between AGA and SD with respect to bacterial or fungal colonization. In contrast, Demodex spp. was significantly more prevalent in AGA, in agreement with previous studies demonstrating an association between Demodex density and advanced AGA, predominantly based on non-invasive imaging methods.^40^ In contrast to reports suggesting an absence of Malassezia spp. in scarring alopecias,^37^ fungal elements were rarely identified in the present cohort, highlighting the limited diagnostic utility of microbial detection for differentiating scarring from non-scarring alopecias.

This study has limitations. The relatively small sample size restricts generalizability, particularly for rare histopathologic findings. The inclusion of male patients only ensured sample homogeneity but limits extrapolation to female populations. The cross-sectional design precludes assessment of temporal relationships between inflammation, fibrosis, and disease progression. Finally, the absence of FAPD biopsy specimens prevents direct histopathologic comparison, and interobserver variability was not assessed.

## Conclusions

This study pioneers the comparative evaluation of transverse scalp biopsy sections from 56 patients with non-scarring scalp disorders, providing novel histopathological insights into their overlap with Fibrosing Alopecia in a Pattern Distribution (FAPD). Perifollicular fibrosis was a relatively frequent finding, challenging traditional distinctions between scarring and non-scarring processes. Perifollicular inflammation was common in both conditions and may mimic lichenoid patterns, representing a potential diagnostic pitfall. In contrast, necrotic keratinocytes were uncommon and not discriminatory. Certain features showed group-specific associations, including basal layer vacuolization and eccrine duct dilatation with AGA, and polytrichia with SD. Follicular miniaturization emerged as a reliable distinguishing feature of AGA, while increased *Demodex sp* density supported its possible proinflammatory role. Overall, these findings reinforce the need for careful clinicopathological correlation to accurately differentiate non-scarring alopecias and conditions from FAPD, thereby avoiding misdiagnosis and ensuring appropriate management and prognostic assessment.

## ORCID ID

Almut Böer-Auer: 0000-0001-7928-8569

Betina Werner: 0000-0002-9671-5603

## Ethics approval statement

This study was approved by the Research Ethics Committee, the Hospital de Clínicas (Federal University of Paraná), Curitiba – Paraná. Certificate of Presentation for Ethical Appreciation (CAAE) 75757223.4.0000.0096, and approval number 6.624.101.

## Financial support

None declared.

## Authors’ contributions

Tatiane Elen Souza: The study concept and design; Data collection, analysis and interpretation of data; Writing of the manuscript; Critical Review of the Literature; Final approval of the final version of the manuscript.

Almut Böer-Auer: Analysis and interpretation of data; Critical review of important intellectual content; Final approval of the final version of the manuscript.

Betina Werner: The study concept and design; Writing of the manuscript and critical review of important intellectual content; Effective participation in the research guidance; Final approval of the final version of the manuscript.

All authors discussed the results and contributed to the final version of the manuscript.

## Research data availability

The entire dataset supporting the results of this study was published in this article.

## Conflicts of interest

None declared.
